# Epidemiology of tobacco use and dependence in adults in a poor peri-urban community in Lima, Peru

**DOI:** 10.1186/1471-2466-12-9

**Published:** 2012-03-19

**Authors:** Paul Logan Weygandt, Elisa Vidal-Cardenas, Robert H Gilman, Erika Avila-Tang, Lilia Cabrera, William Checkley

**Affiliations:** 1 Division of Pulmonary and Critical Care, School of Medicine, Johns Hopkins University, Baltimore, MD, Maryland, USA; 2 Program in Global Disease Epidemiology and Control, Bloomberg School of Public Health, Johns Hopkins University, Baltimore, MD, Maryland, USA; 3 A.B. PRISMA, Lima, Peru; 4 Institute for Global Tobacco Control, Bloomberg School of Public Health, Johns Hopkins University, Baltimore, MD, Maryland, USA

## Abstract

**Background:**

Tobacco smoking is an important public health concern worldwide leading to both chronic disease and early death. In Latin America, smoking prevalence is estimated at approximately 30% and prior studies suggest that the prevalence in Peru is 22% to 38%. We sought to determine the prevalence of daily smoking in a poor peri-urban community in Lima, Peru.

**Methods:**

We conducted a cross-sectional survey in a random sample of adults ≥40 years of age living in Pampas de San Juan de Miraflores, Lima, Peru. We asked participants to respond to a survey that included questions on sociodemographics, tobacco use and dependence.

**Results:**

We enrolled 316 participants. Average monthly household income was ≤ 400 USD and nearly all homes had running water, sewage, and electricity. Most individuals had not completed high school. Smoking prevalence was 16% overall, yet daily smoking prevalence was 1.9%. Former daily smokers comprised 3.8% of current nonsmokers and 9.1% current occasional smokers. Average scores for the Fagerstrom Test for Nicotine Dependence for daily smokers and occasional smokers were 1.5 and 0, respectively.

**Conclusions:**

Daily use of tobacco is uncommon among adults in peri-urban communities of Lima, Peru, unlike their counterparts in Lima and other Latin American capital cities. Tobacco dependence is also low. Hence, efforts aimed at primary prevention are of utmost importance in these communities. This study provides an accurate baseline using an internationally recognized assessment tool (Global Adult Tobacco Survey), allowing for accurate assessment of tobacco control interventions over time.

## Background

Tobacco use is the leading cause of preventable death in the world, resulting in millions of deaths annually, more than HIV/AIDS, tuberculosis and malaria [1]. Tobacco smoking is an important public health concern worldwide leading to pulmonary disease, various cancers including those of the respiratory, digestive, and genitourinary systems, and certain forms of leukemia and premature death [2]. Tobacco smoking causes over half of all avoidable deaths worldwide [3]. It accounted for an estimated four to five million deaths per year by 2000 [4], and contributed to an estimated 4.1% of years of life lost [5]. Low- and middle-income countries comprise 82% of the world smoking population, consume 74% of the total number of inhaled tobacco products consumed each year, and are suffering an increasing proportion of tobacco-related deaths [6]. A recent report from the World Health Organization provides age-standardized estimates of smoking prevalence in Latin America ranging from 14% to 38%, with Belize smoking the least and Chile smoking the most, while data on Peru are not available [7].

Socioeconomic factors influence tobacco consumption worldwide [8]. In the developed world, a strong inverse relationship between socioeconomic status and smoking exists such that the poorest and least educated populations are more likely to smoke [9-12]. While the few studies characterizing tobacco use in the developing world are equivocal, they generally support the findings observed in the developed world [9]. Moreover, it has been suggested that tobacco use has become widely prevalent in developing countries and the public health significance of smoking-related morbidity and mortality will continue to grow [9]. As the tobacco epidemic shifts from developed to developing countries, countries like Peru are in an ideal position for primary intervention programs prior to the realization of full range of smoking-related morbidity and mortality.

Peru has recently transitioned to a rapidly growing upper middle-income country [13]. Chronic disease profiles for many developing countries in transition are substantially and rapidly worsening [14,15]. Peru might well be expected to follow such an epidemiological transition involving a decreasing proportion of infectious diseases and an increasing proportion of chronic diseases. However, data on mortality and detailed studies of smoking are sparse in many developing countries where smoking has already become epidemic [9]. As a country in transition, accurate characterization and quantification of Peru's tobacco burden and use patterns is of public health importance. Previous studies have shown overall smoking prevalence in Peru at 22% to 38% and a recent report in 2005 from the Center for Information and Education for the Prevention of Drug Abuse found prevalence of daily and occasional smoking in urban Peru to be 8% and 20%, respectively [16-21]. However, study methodologies varied greatly and none focused on the low-income, peri-urban shantytowns. These communities that comprise a large portion of the population are less well studied because they are more difficult to access than their urban counterparts. Moreover, these communities are often associated with poor health infrastructure and receive little public-health attention from the government.

While estimates of tobacco smoke prevalence are available for multiple studies in Latin America, few of these studies have been conducted across multiple countries using standardized approaches. Two multi-country studies that used standardized questionnaires found that prevalence for tobacco smoke in Latin America ranging from 24% to 39% in one study [16] and from 22% to 45% in the other [22]. These studies, however, appear to have sampled populations that are either more urbanized [16] or with an overall higher educational level [22] than would be expected from peri-urban communities, which represent a larger share the urban populations in many Latin American cities.

In this study, we sought to characterize the prevalence of smoking in adults aged ≥ 40 years living in a peri-urban shantytown in Lima, Peru. We chose this age group to compare with two recent large-scale studies on COPD, which included tobacco use [16,22]. As a secondary objective, we sought to determine factors influencing tobacco use and dependence. Our primary hypothesis was that the prevalence of daily smoking in this population will be similar to rates observed in prior studies and that those rates would reflect those observed in other Latin American capital cities.

## Methods

### Study setting

We conducted our study in Pampas de San Juan de Miraflores, a poor peri-urban community located approximately 25 kilometers to the southwest of Lima, Peru. The community of Pampas de San Juan de Miraflores was settled in the early 1980s as thousands of poor families migrated into the area as part of the "urban invasion" that began in the 1950s. The state also encouraged urban expansion by encouraging state employees to seek new residence in this area. However, the majority of 72,000 inhabitants of Pampas are highland immigrants.

### Study design

We sought to enroll a representative group of 300 individuals aged ≥ 40 years from census data obtained by A.B. PRISMA in 2002. Recent studies suggested a smoking prevalence in Lima of 27% [16]. Based on a prevalence of 27% and a 0.05 level of confidence, we calculated a minimum sample size of 303 participants. We chose to enroll adults in this age group because they are likely to have the highest risk of developing COPD and because we could directly compare our results against two previous large-scale prevalence studies [16,22] After selecting a simple random group of participants from the census list, field workers traveled to the homes of selected individuals to invite those individuals to participate. We did not include the following individuals in the study: non-residents of Pampas, those who were not part of the census, those younger than 40 years of age, those who refused to participate, those who could not or would not complete both modules of the questionnaire in our study; and those who were selected but could not be located. We enrolled only one person per household to avoid household-level correlation. If an individual either refused to participate or was not met after three visits, we selected another individual from the list. All participants went through the process of informed consent followed by the surveys. The study was approved by the Internal Review Board of A.B. PRISMA in Lima, Peru.

We used questions from the Global Adult Tobacco Survey (GATS), which has been applied in both Mexico and Brazil [23-25], to address both quantity and frequency of tobacco use, access to tobacco, associated costs, personal attitudes toward tobacco, and tobacco dependence. In contrast to the surveys used in BOLD and PLATINO, GATS does not ask if a participant smoked > 100 cigarettes in their lifetime. To evaluate socioeconomic status, we asked about household size, assets, income, and education. We conducted a pilot study prior to enrollment and determined that there was no tobacco use other than cigarettes. Upon enrollment, the field worker interviewed each participant to obtain answers for all questions in our questionnaire.

### Measures

The primary outcome of this study was smoking prevalence. We classified participants into three categories: daily smokers (≥1 cigarette/day), occasional smokers (< 1 cigarette/day), and nonsmokers. We defined former smokers as those who were either current occasional smokers or nonsmokers who smoked daily (≥1 cigarette/day) in the past. Smoking dependence was analyzed using the Fagerstrom Test for Nicotine Dependence [26]. The Fagerstrom Test for Nicotine Dependence has been shown to be highly reliable and it has been shown to correlate with cotinine and exhaled carbon monoxide levels [27-29]. A score of less than four was considered minimal dependence.

### Biostatistical methods

To address our primary objective, we calculated 95% confidence intervals around prevalence estimates of tobacco smoke using standard methods [30]. To address secondary objectives, we calculated simple proportions or means and used multivariable logistic regression to identify important demographic and socioeconomic predictors of smoking. We used R (http://www.r-project.org) for statistical analysis.

## Results

### Baseline characteristics

To obtain a sample size of approximately 300, we attempted to contact a random sample of 683 possible participants between August 2009 and February 2010. 84 (12%) refused the interview; 150 (22%) had moved to a different community or were traveling; 97 (14%) were difficult or impossible to find; and, 33 (5%) had died. In total, we interviewed 319 individuals from August 2009 through February 2010; however, 3 were < 40 years old and were thus excluded. We interviewed a total of 149 men and 167 women for a total of 316 participants. Monthly income ranged between 480 and 1120 soles per month (approximately USD 165 to 385) and the vast majority of homes had electricity, running water and a primary sewage connection. Sixty-one percent (n = 194) had not completed high school. Approximately 1% of participants (n = 4) were unemployed and one person was retired. The remainder of the population was employed in retail, were stay-at-home parents, performed manual labor, worked as private employees, provided domestic services, worked in transportation, were government employees, or had some other source of income (Table 1).

**Table 1:** Baseline characteristics

Characteristic	N (n = 316)	%
Sex		

Male	149	47%

Female	167	53%

Age		

40-49	129	41%

50-59	140	44%

60-85	47	15%

Race		

Mestizo	308	98%

Black	6	2%

White	2	< 1%

Education		

No school/illiterate	11	3%

Primary incomplete or no school/literate	59	19%

Primary complete	124	39%

Secondary complete	77	24%

Technical school, university, or postgraduate education undertaken	45	14%

Primary Work		

Private or government employee	42	13%

Retail	70	22%

Service - manual labor, transportation, or domestic	106	34%

Stay at home parent	68	22%

Retired or unemployed	5	2%

Other	24	8%

Water		

Running water in home	301	95%

Well inside home	3	1%

Other (e.g., public well/pipe/tank, water delivery, spring, etc.)	12	4%

Sanitation		

Toilet with sewage	308	97%

No toilet/sewage	8	3%

Electricity		

Yes	314	99%

No	2	1%

Income		

< 480 soles	31	10%

481-800 soles	109	34%

801-1120	97	31%

1121-1440	36	11%

≥ 1441 soles	26	8%

Number of families in the household		

1	247	78%

2	53	17%

≥ 3	16	5%

Number of rooms used for sleeping		

1	23	7%

2	77	24%

3	120	38%

≥ 4	95	30%

### Tobacco use and dependence

The overall prevalence of smoking in our study group, defined as either occasional or daily use, was 16% (25% in men and 7.8% in women); however, only 1.9% of participants (3.4% in men and 0.6% in women) were daily smokers (Table 2). Of current nonsmokers and occasional smokers, 3.8% and 9.1%, respectively, were former daily smokers (eTable 1). While the overall prevalence of smoking was similar to those observed in other Latin American countries, the number of daily smokers in our study was very low (Table 3).

**Table 2:** Prevalence of current tobacco use stratified by sex and age

	Number of men (%)			Number of women (%)			Overall (%)		
**Age (years)**	**Nonsmokers**	**Occasional smokers**	**Daily Smokers**	**Nonsmokers**	**Occasional Smokers**	**Daily Smokers**	**Nonsmokers**	**Occasional Smokers**	**Daily Smokers**

40-49	40 (71%)	15 (27%)	1 (2%)	66 (90%)	6 (8%)	1 (4%)	106 (82%)	21 (16%)	2 (2%)

50-59	49 (73%)	15 (22%)	3 (5%)	67 (92%)	6 (8%)	0 (0%)	116 (83%)	21 (15%)	3 (2%)

60-85	23 (88%)	2 (8%)	1 (4%)	21 (100%)	0 (0%)	0 (0%)	44 (94%)	2 (4%)	1 (2%)

Total	112 (75%)	32 (22%)	5 (3%)	154 (92%)	12 (7%)	1 (1%)	266 (84%)	44 (14%)	6 (2%)

**Table 3:** Comparison of daily smoking prevalence in Lima, Peru and other Latin American countries and capital cities

Study	Location	Year Conducted	Age	Prevalence measure	Prevalence	95% CI
This study	Lima, Peru	2009	≥40	Current daily smokers	1.9%	(0.8 to 4.3)

Epidemiologia de drogas en la población urbana peruana 2005 [17]	Lima, Peru	2005	12-64	Current daily smokers	7.7%	Not reported

CARMELA [16]	Lima, Peru	2003-2005	25-44	Current smokers	26.6%	(23.9-29.4)

II Encuesta nacional sobre prevención y consumo de drogas 2002 [19]	Peru	2002-2003	41-64	Annual prevalence	25-34%	Not reported

GATS [25]	Brazil	2008	> 15	Current daily smokers	15.1%	(14.6-15.5)

GATS [25]	Brazil	2008	≥15	Current smokers	17.1%	(16.6-17.6)

GATS [24]	Mexico	2009	≥15	Current daily smokers	7.6%	(6.8-8.3)

GATS [24]	Mexico	2009	≥15	Current smokers	15.9%	(14.7-17.1)

PLATINO [22]	Sao Paulo	2003	≥40	Current smokers	23.9%	(21.3 to 26.6)

PLATINO [22]	Santiago	2004	≥40	Current smokers	38.5%	(35.7 to 41.2)

PLATINO [22]	Mexico City	2003	≥40	Current smokers	25.4%	(22.8 to 28.0)

PLATINO [22]	Montevideo	2004	≥40	Current smokers	28.0%	(25.2 to 30.9)

PLATINO [22]	Caracas	2004	≥40	Current smokers	28.5%	(26.1 to 30.9)

Among current daily smokers (n = 6), smoking began at a mean age of 18 years (SD = 4.7). Among previous daily smokers, smoking began at a mean age of 27 years (SD = 11). Current daily smokers smoked for a mean of 34 years (SD = 12) and were exposed to a mean of 8.2 (SD = 6.2) pack-years of tobacco use. Previous daily smokers smoked for a mean of 18 years (SD = 12). We calculated average Fagerstrom Test for Nicotine Dependence scores by frequency of smoking. Occasional smokers averaged a score of 0, while the five daily smokers who responded averaged a score of 1.5 (eTable 2).

Men were five times more likely to smoke than women (OR = 4.97, 95% CI 2.3 to 10.7). Participants with households where smoking was never allowed were six times less likely to smoke than participants in households where smoking was allowed (OR = 0.2, 95% CI 0.1 to 0.4). Income was not associated with smoking status at the 0.05 level of significance. Neither were having assets such as a car or fridge, or being married or in a committed relationship (all p > 0.05). Seventy-two percent (n = 227) of study participants had seen cigarettes being sold on the streets in the last 30 days. Twenty-eight percent (n = 90) reported having bough cigarettes for others. Seventy-one percent (n = 63) bought cigarettes for others less than once a month. Seventy-two percent (n = 227) reported having observed cigarettes being sold on the street.

### Secondhand smoke

Smoking was never allowed in 47% (n = 125) of the homes of nonsmokers, whereas smoking was allowed within the homes of 100% (n = 6) of current daily smokers. Three percent of nonsmokers (n = 4) allowed smoking on a daily basis within the home, while 67% of current daily smokers (n = 4) allowed daily smoking within the home. During the previous 30 days, 28% of nonsmokers (n = 26) reported someone smoking indoors at their workplace, while 75% of current daily smokers (n = 3) reported smoking in the workplace; and, 23% of respondents (n = 39) indicated they had observed someone smoking in restaurants and 19% of respondents (n = 56) reported observing smoking in public transportation (Table 4).

**Table 4:** Secondhand smoke, media, advertisement and beliefs about tobacco

SECONDHAND SMOKE			
	**Nonsmokers (%)**	**Occasional smokers (%)**	**Daily smokers (%)**

**Smoking house rules?**			

Never allowed	125 (47%)	7 (16%)	0 (0%)

Allowed but exceptions	133 (50%)	31 (72%)	4 (67%)

Permitted	4 (2%)	4 (9%)	2 (33%)

No rules	4 (2%)	1 (2%)	0 (0%)

**How often does someone smoke inside your house?**			

Less than monthly	125 (89%)	30 (86%)	1 (17%)

Monthly	3 (2%)	3 (9%)	1 (17%)

Weekly	9 (6%)	2 (6%)	0 (0%)

Daily	4 (3%)	0 (0%)	4 (67%)

**Workplace policy?**			

Not allowed	75 (78%)	19 (86%)	4 (100%)

Allowed in some areas	11 (11%)	2 (9%)	0 (0%)

Allowed anywhere	7 (7%)	1 (5%)	0 (0%)

There is no policy	3 (3%)	0 (0%)	0 (0%)

**Did anyone smoke indoors where you work in the last 30 days?**			

No	68 (72%)	18 (78%)	1 (25%)

Yes	26 (28%)	5 (22%)	3 (75%)

**Did anyone smoke inside government buildings in the last 30 days?**			

No	45 (79%)	10 (77%)	0 (0%)

Yes	12 (21%)	3 (23%)	1 (100%)

**Did anyone smoke inside health care facility in the last 30 days?**			

No	105 (95%)	14 (100%)	2 (67%)

Yes	6 (5%)	0 (0%)	1 (33%)

**Did anyone smoke inside of restaurants in the last 30 days?**			

No	101 (75%)	27 (84%)	3 (75%)

Yes	33 (25%)	5 (16%)	1 (25%)

**Did anyone smoke inside of public transport in the last 30 days?**			

No	204 (81%)	36 (86%)	5 (83%)

Yes	49 (19%)	6 (14%)	1 (17%)

**MEDIA, ADVERTISEMENT AND BELIEFS ABOUT TOBACCO**			

	**Yes (%)**	**No (%)**	**Did not respond or Don't know (%)**

**Has interviewee seen advertisements for cigarettes in the last 30 days ...**			

Stores	163 (52%)	150 (47%)	3 (1%)

Television	168 (53%)	144 (46%)	4 (1%)

Radio	128 (41%)	182 (58%)	6 (2%)

Posters/billboards	167 (53%)	134 (42%)	15 (5%)

Newspapers/magazines	155 (49%)	144 (46%)	17 (5%)

Cinema	11 (3%)	95 (30%)	210 (66%)

Internet	8 (3%)	109 (34%)	199 (63%)

Public transport vehicles/stations	146 (46%)	161 (51%)	9 (3%)

Public walls, signposts, or banners	119 (38%)	181 (57%)	14 (4%)

Sporting events	13 (4%)	299 (95%)	4 (1%)

**Has interviewee seen promotions for cigarettes in the last 30 days ...**			

Free cigarettes?	33 (10%)	283 (90%)	0 (0%)

Cigarettes at sale prices?	43 (14%)	269 (85%)	4 (1%)

Coupons?	20 (6%)	294 (93%)	2 (1%)

Free gifts, discounts on other products?	23 (7%)	290 (92%)	3 (1%)

Clothing or other items with brand or logo?	63 (20%)	252 (80%)	1 (< 1%)

Promotions by mail?	2 (1%)	116 (37%)	198 (63%)

**Does interviewee believe cigarette smoking causes serious illness?**			

Men	146 (98%)	3 (2%)	0 (0%)

Women	166 (99%)	1 (1%)	0 (0%)

**Does interviewee believe cigarette smoking causes stroke?**			

Men	115 (77%)	27 (18%)	7 (5%)

Women	136 (81%)	19 (11%)	12 (7%)

**Does interviewee believe cigarette smoking causes heart attack?**			

Men	127 (85%)	15 (10%)	7 (5%)

Women	153 (92%)	9 (5%)	5 (3%)

**Does interviewee believe cigarette smoking causes lung cancer?**			

Men	145 (97%)	1 (1%)	3 (2%)

Women	166 (99%)	0 (0%)	1 (1%)

### Media and advertisement

Between 38% and 53% of participants reported having observed advertisements for cigarettes in stores, on television, on the radio, on posters or billboards, and in newspapers or magazines, in public transport vehicles, and on public walls, signposts, or banners in the previous 30 days. Between 3% and 4% of interviewees observed advertisements for cigarettes at the cinema, on the Internet, or at sporting events within the previous 30 days. A small number of participants had seen promotions such as free cigarettes, cigarettes at sale prices, coupons, free gifts, discounts on other products, clothing or other items with the brand or logo, or received any promotions by mail (Table 4).

### Beliefs about tobacco

98% of men and 99% of women indicated that they believed that cigarette smoking causes serious illness. Similarly 97% of men and 99% of women indicated that they believed cigarette smoking causes lung cancer. 85% of men and 92% of women indicated that they believed cigarette smoking causes heart attack and fewer still, 77% of men and 81% of women, indicated that they believed cigarette smoking causes stroke (Table 4).

## Discussion

In our study we observed a low prevalence of daily smoking in adults 40 years and older living in Pampas de San Juan de Miraflores, a poor peri-urban shantytown in Lima, Peru. Results from the Fagerstrom Test for Nicotine Dependence survey indicated minimal dependence even among current daily smokers thus supporting a low tobacco burden in this community. While the Fagerstrom Test for Nicotine Dependence survey may be less applicable in populations with low levels of tobacco use, our results suggest that even in those few regular smokers that were studied, physical dependence is minimal. Our study cannot generalize about smoking prevalence in more affluent populations living in urban areas of Lima; however, poor, peri-urban communities like Pampas de San Juan represent > 50% of the population in Lima, Peru. The low prevalence of smoking found in our community underscores the importance of early anti-tobacco campaigns as a primary prevention strategy.

Recent studies have shown current smoking rates of 27% in Lima, which is substantially higher than the rates we observed [16,17]. The disparity between values for smoking prevalence we observed in Pampas de San Juan de Miraflores and those observed in previous studies may partially stem from differences in the manner in which smokers were classified in each study. Information regarding methods of determining smoking status was unclear in several prior studies of smoking in Peru [19,31]. Other studies have been more explicit. The Center for Information and Education for the Prevention of Drug Abuse focused on urban centers. They reported lifetime prevalence, but broke down smokers into those who smoked daily, those who smoked at least once per week, and those who smoked less than once a week but greater than once a month [17,18]. To classify subjects into smoking categories, the PLATINO group implemented the Lung Health Study Questionnaire; and classified individuals who had smoked in the previous 30 days as current smokers. The PLATINO study found < 1% of the individuals identified current smokers reported not to be daily smokers [22]. In contrast, 88% of the individuals we observed in our study to be smokers were not daily smokers. The CARMELA study classified individuals as current smokers if they were current daily or occasional smokers with a lifetime smoking history of at least 100 cigarettes [16]. Standardized methods for collecting and reporting data on smoking status, such as those employed by the Global Adult Tobacco Survey and CARMELA groups will help to provide a clearer picture of the burden of tobacco smoking worldwide. We suggest that using a cutoff of at least one cigarette per day is a more appropriate measure of current smoking status and avoids overestimation that may occur by simply asking participants if they had smoked in the last 30 days or had a history of a lifetime consumption of ≥100 cigarettes. Despite slight differences in our approach to the quantification of smoking, the number of current daily smokers in our study was remarkably low.

The low smoking prevalence we observed in adults ≥ 40 years of age in Pampas San Juan de Miraflores is likely due to several factors that influence smoking behaviors that need to be investigated further. It has been previously suggested that providing health information and prominent warning labels are highly relevant tobacco control policies for low-middle income countries [32]. The vast majority of the study population understood that smoking could cause serious health problems including lung cancer, heart attacks, and cerebrovascular accidents. While education is important in reducing the burden of tobacco use, the economics of tobacco consumption likely serves as the principal determinant of tobacco smoking behaviors. San Juan de Miraflores is an economically depressed community, with a household income of less than 400 USD per month. In populations with low socioeconomic status, individuals are more responsive to changes in prices of cigarettes (Townsend et al., 1994). It has been suggested that price increases of 10% would be the most cost effective intervention for reducing smoking prevalence and smoking-related mortality and that price interventions would be particularly effective in low and middle-income countries [32,33]. Hence, taxation in developing countries would likely lead to decreased smoking prevalence in poor peri-urban communities. Moreover, as income levels and development continues to improve communities like Pampas de San Juan de Miraflores, longitudinal studies tracking tobacco costs versus smoking prevalence in a representative group of the population would likely clarify the relationship between economics and smoking in this community.

The majority of people observed cigarettes being sold on the streets. These are often sold as single cigarettes or packs of five cigarettes. As cigarettes sold on the street are cheaper than large packs and more accessible, this is likely an area for effective policy intervention. By eliminating the sale of cigarettes on the street, the already low tobacco burden in this population could likely be further reduced. Moreover, cigarettes sold on the street are more accessible to young individuals and thus intervention might help reduce the incidence of smoking in this community.

The majority of study participants observed the sale of tobacco products on the street. Strategies aimed at preventing the sale of cigarettes on the street would likely lead to a reduction of smoking prevalence and incidence. While many strategies may be employed to prevent the initiation of smoking, we suggest that education, taxation, efforts to curtail the sale of cigarettes on the streets, and strict enforcement of recent laws prohibiting smoking in eating and drinking establishments, government buildings, and public transportation would be particularly beneficial.

By focusing on primary prevention, governmental and public health organizations can help maintain a low smoking prevalence in poor, peri-urban communities in Lima, Peru. Further epidemiological studies in these populations may yield important information suggesting the reasons behind the low prevalence of smoking. We also suggest the need for repeat surveys over time to assess changing incidence and prevalence with the implementation of new tobacco control policies. These longitudinal studies may well elucidate the factors leading to the remarkably low smoking prevalence in this community and may offer new strategies to be implemented on the international stage.

A limitation of this study is that data were obtained via self-report. In the future use of quantitative measures of tobacco exposure such as exhaled carbon monoxide, urine or saliva cotinine, or hair nicotine would allow us to more definitively characterize tobacco exposure in this population. However, self-reported smoking status is the primary measure of smoking status for research and policy, and it provides both a cheaper and widely accepted indicator. Another limitation of this study is its cross-sectional nature. Longitudinal studies of this population in the future might yield insight into trends in tobacco prevalence and incidence and the outcomes of current and future tobacco control measures. Third, we focused on a group of adults ≥ 40 years of age and did not include adolescents and younger adults who may be at a high of risk of tobacco use. While younger people may be smoking at greater rates and have younger ages of onset, we were interested in first estimating the current level of pack-years which requires a long history of smoking; and, second, we wanted to compare the prevalence of smoking with the two large-scales studies PLATINO and CARMELA. This study was a pilot to better understand tobacco use and dependence in adults, the segment of the population most at risk for tobacco-related lung disease. Our group is conducting a 3000-person study in adults ≥ 35 years of age to determine lung function decline and we sought to find the best epidemiological measure of tobacco use in our region.

Our study has several strengths. First, we obtained adults ≥ 40 years were randomly selected. Therefore, we are confident that our sample is representative of the study population and largely representative of the population in Lima. Second, we used the well-validated Global Adult Tobacco Survey and Fagerstrom Test for Nicotine Dependence, both which provide a standardized set of questions that can be directly comparable with other studies. Third, we surveyed a relatively large number of male and female participants so we are confident that the low prevalence of smoking is not due to a small sample size. Finally, we do not believe that our findings are attributable to low reporting. In previous studies, self-reporting smoking in adults has been shown to be accurate in most studies [34,35].

In the midst of this major shift in Peru's public health paradigm, we are in a unique position to complete the research needed to provide data critical to shaping tobacco-related policy. Thus, more surveys such as the one in this study are needed to implement the provisions of the International Convention on Tobacco Control in developing countries. Moreover, standardization of survey techniques allows for comparison between various tobacco control interventions in order to yield consistent data upon which public health decisions can be made. These data are of paramount importance if lawmakers, public health workers, and health care professionals are to enact effective policy for reducing the burden of tobacco consumption with its concomitant disease and disability.

## Conclusions

Using an internationally recognized assessment tool, we found that the prevalence of daily smoking was remarkably low in a peri-urban community in Lima, Peru, when compared with other populations in Latin America. There was a concomitant low prevalence of nicotine dependence in our study population. Despite slight differences in methodologies between our study and others, there was a dramatically lower prevalence of daily smoking in our study population than in other Latin American cities. While many factors influence tobacco consumption, tobacco economics and public education are likely two of the most important factors associated with remarkably low smoking prevalence observed in this study. The low prevalence of smoking found in our community underscores the importance of early anti-tobacco campaigns as a primary prevention strategy.

### What this paper adds

Smoking is an important public health issue worldwide. Previous studies have estimated smoking prevalence in Peru to be 22% to 38%; however, studies have differed in their methods and definitions of smoking status, and none have focused specifically on the poor peri-urban shantytowns that comprise a large proportion of Peru's population. This study reports that smoking prevalence in Pampas de San Juan de Miraflores, a poor peri-urban shantytown in Lima, Peru, is strikingly low. Nicotine dependence in this community is also remarkably low. Our results suggest that tobacco economics and public education may play an important role in influencing tobacco-smoking habits.

## Competing interests

The authors declare that they have no competing interests.

## Authors' contributions

PLW and WC were responsible for study design, analysis of data, and writing of manuscript. EVC participated in coordination and writing of manuscript, LC participating in design, coordination and data collection and writing of manuscript, RHG assisted in the design and writing of manuscript, and EAT contributed to the design and writing of the manuscript. All authors read and approved the final manuscript.

## Sources of funding

This work was supported by Asociación Benéfica PRISMA. P. Logan Weygandt was supported in part by the Johns Hopkins University School of Medicine's Summer Research Program and the Andrew Watson Sellards Fund for the analysis of these data. William Checkley was supported by a Clinician Scientist Award from the Johns Hopkins University and a K99/R00 Pathway to Independence Award (R00HL096955) from the National Heart, Lung and Blood Institute, National Institutes of Health. William Checkley and Robert Gilman were further supported by a contract (HHSN268200900033C) with the National Heart, Lung and Blood Institute, National Institutes of Health.

## Pre-publication history

The pre-publication history for this paper can be accessed here:

http://www.biomedcentral.com/1471-2466/12/9/prepub
